# Editorial: New Perspectives in Psychopathology

**DOI:** 10.3389/fpsyt.2019.01009

**Published:** 2020-02-03

**Authors:** Diogo Telles Correia, Elie Cheniaux

**Affiliations:** ^1^ Department of Psychiatry, University of Lisbon, Lisbon, Portugal; ^2^ Department of Psychiatry, Federal University of Rio de Janeiro, Rio de Janeiro, Brazil

**Keywords:** psychopathology, psychiatric classifications, psychiatric disorders, psychiatric symptoms, research in psychopathology

## Where Does Research in Psychopatology Stand?

On the one hand, current psychiatric classifications do not fit the needs of clinicians, (with many psychopathological syndromes not falling into any current classificatory category, for example) (1).

On the other hand, current classifications fail even more in relation to research, namely translational research. New classifications that seek a paradigm shift (Rdoc) have been proposed to solve this last issue (2), but are also criticized because they imply a blurring of the clinical reality of the symptom that defines mental illness to a notion centered on the few neurobiological correlates of some mental activities (3). New insights assert that there may be a need for the co-existence of various classifications, some more used in research and others more suited to clinical practice and decision making (3).

The methodology used to search for neurobiological correlates of psychopathological manifestations is still quite unsatisfactory as well. There is an epistemological gulf between clinical psychopathological and neurobiological assessment, and correlations between these two realities are difficult to establish(4–6).

Additionally, much remains to be known regarding causal models in psychopathology. Indeed, the role of biological factors in the development of mental disorders, as well as the role of environmental factors (namely life events), and more than the individual contribution of each factor, its integrative multicausal model, has yet to be established (4). Current causal models do not adequately explain psychopathological manifestations, and are unsatisfactory for use in research. Accepting the fact that it may be difficult or even impossible to find a definitive model, new proposals that are increasingly appropriate and useful to the clinical and research reality are urgent.

This Research Topic attempts to aggregate several contributions that reflect the new insights that have emerged in research in psychopathology.

Within this Research Topic we had 28 contributions, 15 original research articles, five review articles, seven perspective articles, one hypothesis and theory article.

We sumarize this contributions below.

Regarding the original research, in the paper entitled “ERP Evidence for Inhibitory Control Deficits in Test-Anxious Individuals”, forty-six participants were recruited and divided into a HTA (N = 26) and low test anxiety (LTA; i.e., healthy control; N = 20) group. Self-reports (Test Anxiety Scale, State-Trait Anxiety Inventory for negative emotions) were obtained. An emotional Stroop (ES) task and a numerical Stroop (NS) task, causing different types of interferences, were used for assessing the emotional and cognitive aspects of attentional control ability (behavioral data). The authors concluded that high test anxiety individuals have extensive inhibitory deficits for both emotional and cognitive aspects; however, impairment impacts are more on emotional aspects than on cognitive aspects (Zhang et al.).

In “Early Trauma and Cognitive Functions of Patients With Schizophrenia”, the authors aimed to investigate the putative correlation between early trauma and cognitive functions, and also bettween psychotic symptoms and cognitive functions, in patients with schizophrenia. A quantitative assessment was performed with 20 individuals diagnosed with schizophrenia according to the 5th edition of the Diagnostic and Statistical Manual (DSM-5) criteria. Clinical [Positive and Negative Syndrome Scale (PANSS), and the Early Trauma Inventory Self-Report—Short Form (ETISR-SF)] and cognitive measurments [Beta III test, Concentrated Attention (CA) test, Color Trails Test (CTT), and Visual Face Memory (VFM)] were preformed. The authors found that there was an association between early trauma experience and cognitive impairment such as visual memory, as well as a relationship between negative symptoms and attention domains (Carrilho et al.).

In “Associative Memory Impairments Are Associated With Functional Alterations Within the Memory Network in Schizophrenia Patients and Their Unaffected First-Degree Relatives: An fMRI Study”, the authors used an associative memory task to test the hypothesis that SZ patients and first-degree relatives have altered functional patterns in comparison to healthy controls. They found that the findings of first-degree relatives indicated slightly different functional pattern within brain networks in contrast to controls without significant differences in the behavioral task (Oertel et al.).

In “Relationship Between Depression and Subtypes of Early Life Stress in Adult Psychiatric Patients”, the relationship between depression and subtypes of early life stress among 81 psychiatric patients treated at the inpatient Day Hospital Unit of a University General Hospital was studied. It was demonstrated that emotional abuse was a significant risk factor involved in the pathogenesis of depression (Martins-Monteverde et al.).

In “EEG 40 Hz Coherence Decreases in REM Sleep and Ketamine Model of Psychosis”, that concluded that functional interactions between cortical areas in the gamma frequency band decrease in both experimental models of psychosis (Castro-Zaballa et al.).

In “Altered Gamma-Band Activity as a Potential Biomarker for the Recurrence of Major Depressive Disorder”, 33 healthy control participants and 18 participants with major depressive disorder, completed a lexical emotion identification task during electroencephalography along with assessments of cognitive reactivity after negative mood induction. The authors concluded that the major depressive group had significantly higher cognitive reactivity scores than did the control group and that the power of late gamma-band responses to positive words was significantly greater in the this group (Yamamoto et al.).

In “Hypomania Symptoms Across Psychiatric Disorders: Screening Use of the Hypomania Check-List 32 at Admission to an Outpatient Psychiatry Clinic”, the authors tested the psychometric properties of a European Portuguese adaptation of the HCL-32, establishing its factor structure, reliability, and construct validity. It was concluded that the HCL-32 can be used as a screening tool for Bipolar Spectrum Disorders among adult patients presenting in an outpatient psychiatric clinical setting (Camacho et al.).

In “Eating Disorders Impact on Vigilance and Decision Making of a Community Sample of Treatment Naive Attention-Deficit/Hyperactivity Disorder”, 90 college students arranged in three groups [Attention-Deficit/Hyperactivity Disorder (ADHD)+Eating Disorder (ED), ADHD only and Controls] were analyzed using semi-structured interviews for ADHD (K-SADS), the Iowa Gambling Task, the Conner’s Continuous Performance Test, Digit and Visual span, as well as rating scales for anxiety (STAI), depression (BDI) and impulsivity (BIS-11), and binge eating (BES). It was found that the presence of an ED in normal weight in a community sample of ADHD individuals is associated with higher body mass index and a worse cognitive functioning (Nazar et al.).

In “White Matter Microstructural Changes and Episodic Memory Disturbances in Late-Onset Bipolar Disorder”, diffusion tensor imaging (DTI) and volumetric measures were carried out in early-onset bipolar patients (EOD) (*n* = 16), late-onset bipolar disorder (LOD) (*n* = 14) and healthy controls (*n* = 32). Authors demonstrated that LOD was associated with more extensive WM microstructural changes and worse episodic memory performance than EOD (Late-Onset Bipolar Disorder) (Alves et al.).

In “Criterion Validity of the Yale-Brown Obsessive-Compulsive Scale Second Edition for Diagnosis of Obsessive-Compulsive Disorder in Adults”, the authors intended to test the factor structure and criterion validity of the Y-BOCS-II. For that the Y-BOCS-II and other psychometric instruments, as the OCD subscale of the Structured Clinical Interview for the DSM-IV, were administered to 187 participants (52 patients with OCD, 18 with other mood and anxiety disorders, and 117 healthy subjects). It was concluded this scale has excellent psychometric properties to assess the severity of obsessive-compulsive symptoms, reflecting obsessive, and compulsive dimensions, compatible with currently defined subscales (Castro-Rodrigues et al.).

In “Disconnected—Impaired Interoceptive Accuracy and Its Association With Self-Perception and Cardiac Vagal Tone in Patients With Dissociative Disorder”, 18 patients suffering from dissociative disorders and 18 healthy controls were assessed with the Mental Tracking Paradigm by Schandry for heartbeat detection at baseline and after confrontations exposing them to their own faces in a mirror. The cardiac vagal tone was also assessed. The authors concluded that in the patient group, higher cardiac vagal tone was associated with a more precise heartbeat detection performance and also that dissociative disorder patients showed a considerable deficit in interoceptive accuracy. Hence they argue that therapeutic approaches enhancing interoceptive accuracy and cardiac vagal tone may be considered important and practicable steps to improve the therapy outcome of this patient group (Schäflein et al.).

In “The Effect of Enumeration of Self-Relevant Words on Self-Focused Attention and Repetitive Negative Thoughts”, 146 undergraduate students completed a measure of state anxiety, the SRW enumeration task, Repetitive Thinking Questionnaire, Short Fear of Negative Evaluation Scale, and Rumination-Reflection Questionnaire, before and after imagining a social failure situation.

It was concluded that there was a significant positive effect of the self-relevance of negative SRWs on repetitive negative thinking.

The authors argue that SRW enumeration might enable selective and independent detection of the degree of self-reflection and self-rumination (Murinaka and Sasaki).

In “Psychological Distress Symptoms Associated With Life Events in Patients With Bipolar Disorder: A Cross-Sectional Study”, 79 bipolar patients (depression group, *n* = 32; mania, *n* = 22; euthymia, *n* = 25) were assessed by means of Impact of Event Scale-Revised (IES-R), Hamilton Depression Rating Scale (HDRS), and Young Mania Rating Scale (YMRS). It was found that the HDRS, but not the YMRS, scores showed significant correlations with the IES-R scores (depression group, *r* = 0.42; mania, *r* = 0.64; euthymia, *r* = 0.70). The authors conclude that the depressive symptoms may be closely related to the psychological distress symptoms associated with stressful past events in patients with bipolar disorder (Sato et al.).

In “Altered Resting State Effective Connectivity of Anterior Insula in Depression”, the authors tried to find the differences in effective connectivity among eight right hemisphere brain areas—anterior insula, inferior frontal gyrus, middle frontal gyrus (MFG), frontal eye field, anterior cingulate cortex, superior parietal lobe, amygdala, and hippocampus, between a group of healthy controls (*N* = 20) and medicated depressed patients (*N* = 20).

Authors found that patients had significantly reduced strength of the connection from the anterior insula to the MFG (i.e., dorsolateral prefrontal cortex) and also that there was a significant connection between the amygdala and the anterior insula. These results support and enrich previous data on the role of the right anterior insula in the pathophysiology of depression (Kandilarova et al.).

In “Psychopathology Assessment Methods Revisited: On Translational Cross-Validation of Clinical Self-Evaluation Scale and fMRI”, 18 adult subjects with a depressive episode in the context of major depressive disorder (12 subjects) or bipolar affective disorder (6 subjects) and 18 healthy controls, were studied by means of clinical self-assessment (using Zerssen’s depression scale) and simultaneously administered fMRI. The authors confirmed the possibility of translational cross-validation of a clinical psychological test (von Zerssen’s depression scale) and fMRI (Stoyanov et al.).

Regarding the review articles, in the paper “Mental State Examination and Its Procedures—Narrative Review of Brazilian Descriptive Psychopathology”, searches, interviews, and narrative reviews were done to look for systematic ways in which to conduct mental state examination. It is argued that there might have been a shift from detailed descriptive findings, to an array of observed pathological elements, described through a mental function checklist was observed over time, and that better MSE practices might depend on the recovery of psychopathological debates and semiological reasoning (Rocha Neto et al.).

In “Formal Thought Disorders—Historical Roots”, the authors intended to review the historical roots of Formal Thought Disorders, from the XIX (with Esquirol) century into modern times. The history of this category of psychopathological symptoms is extensively reviewed (including contributes of Esquirol, Kraeplin, Kleist, Bleuler, Kretschemer, Carl Schneider, Goldstein, Cameron, Hamilton, Fish, and Nancy Andreasen) (Jerónimo et al.).

In “Social Cognition in Schizophrenia and Autism Spectrum Disorders: A Systematic Review and Meta-Analysis of Direct Comparisons” a systematic review of literature on Pubmed, Web of Science, and Scopus was preformed including keywords such as “social cognition,” “theory of mind,” “autism,” “Asperger,” “psychosis,” and “schizophrenia.” Data was selected and extracted according to PRISMA guidelines. It was concluded that combining behavioral tasks with neurophysiologic assessments may better characterize the differences in social cognition between both disorders (Fernandes et al.).

In, “Processing of Emotion in Functional Neurological Disorder”, the authors intended to review the evidence of an association between functional neurological disorder and emotions as formulated by Breuer and Freud in their conception of hysterical conversion. They concluded that provide there was some evidence for abnormal bodily awareness in Functional Neurological Disorder. According to their fidings, the authors propose that functional neurological symptoms are forms of emotional reactions shaped into symptoms by previous experience with illness and possibly reinforced by actual social contexts (Sojka et al.).

In, “Culture and Psychopathology: New Perspectives on Research, Practice, and Clinical Training in a Globalized World”, the author discuss the role of culture in understanding and treating psychopathology regarding the inevitable link between psychopathology and culture. New perspectives on the conceptualization of psychopathology and on the definition of culture and how these are intertwined in the implocations of culture in research and clinical training in psychopathology are also approached (Moleiro).

Regarding the perspective articles, in “Seeing Beyond Diseases and Disorders: Symptom Complexes as Manifestations of Mental Constituents”, the author questions whether mental symptom complexes are manifestations of mind constituents or functions that make human experience and mind possible. Several authors are revisited such as Carl Schneider and Kraeplin with this purpose. The authors also expect that worldwide research in this field could include this perspective (Daker).

In “From Affective Science to Psychiatric Disorder: Ontology as a Semantic Bridge”, where the authors propose and discuss an ontological framework for explicitly capturing the complex interrelations between affective entities and psychiatric disorders, in order to facilitate mapping and integration between affective science and psychiatric diagnostics. They argue that this framework is relevant for several purpuses such as clarifying psychiatric diagnostic categories, clinical information systems, and the integration and translation of research results across disciplines (Larsen and Hastings).

In “New Perspectives in Phenomenological Psychopathology: Its Use in Psychiatric Treatment”, two contemporary models for clinical practice based on phenomenological psychopathology are proposed: Dialectical-proportional oriented approach and Person-centered dialectic approach. The first one favors the observation of the complexities inherent to each mode of pathological experience, for instance, schizophrenia can be understood not just from its core elements of delusion, but from the dialectical relationship between the loss of the constitution of reality and its maintenance. The latter one, supports the patient’s unfolding his personal experience and helps him to identify a core-meaning in his experiences around which his narrative can become meaningful (Messas et al.).

In “Mental Ill-Health and the Epidemiology of Representations”, where it is argued that it is possible to uphold the idea of a supra-individual dimension to mental health, while avoiding the obvious pitfalls involved in categorical diagnosing of society as suffering from mental illness. The author defends an extended notion of public mental ill-health, which goes beyond the quantitative understanding of mental health as an aggregate of individual diseased minds captured in statistics, and which can be conceived as a dynamic, emergent property resulting from interactions of individual brains/minds in social space (Kesner).

In “Bleuler’s Psychopathological Perspective on Schizophrenia Delusions: Towards New Tools in Psychotherapy Treatment”, it is intended to highlight Bleuler’s psychopathological contribution to the affective and meaningful causality of delusions in schizophrenia. The role of delusions in the psychopathology of schizophrenia was explored in a close relation with the Bleuler’s fundamental symptoms (Alogia, Autism, Ambivalence, and Affect Blunting), persecutory, grandiosity, and sexual delusions in schizophrenia were also explained invoking Bleulers’ concepts, and described according to the tension between logic and affects, as well as, internal conflict, schizoid features, and auto-erotism as key psychopathological pathways (Arantes-Gonçalves et al.).

In “A Perspective on a Possible Relation Between the Psychopathology of the Schizophrenia/Schizoaffective Spectrum and Unconjugated Bilirubin: A Longitudinal Protocol Study”, it is argued the possibility of a relation between Unconjugated Bilirubin (UCB) plasma high levels and schizophrenia. A study protocol was suggested to investigate this relation. It would be an observational longitudinal study, with two assessments in 1 year time span, in order to achieve a better correlation between variables during the evolution of the patient’s disorder and its respective treatment (Marques and Arantes-Gonçalves).

In, “Mental Disorder—The Need for an Accurate Definition”, a review was preformed about the factors that substantiated the emergence of the first formal definition of mental disorder that based all its later versions. The authors propose that the distress and disability criteria have to be considered in any present and future definitions of mental disorder (Telles-Correia et al.).

Regarding the hypothesis and theory article, in “Gene × Environment Interaction in Developmental Disorders: Where Do We Stand and What’s Next?”, the authors explore the need to move beyond merely examining statistical interactions between genes and the environment, and the motivation to investigate specific genetic susceptibility and environmental contexts that drive developmental disorders.

It is proposed that further parsing of genetic and environmental components is required to fully understand the unique contribution of each factor to the etiology of developmental disorders (Esposito et al.).

## Conclusions

In this Research Topic we have tried to attract new contributions that include different types of research that we consider essential in psychopathology: Review studies and conceptual analysis and original studies.

Psychopathology as we know it today resulted from a long process of conceptual analysis (adapted to the social, cultural, and scientific reality of the time) of 19th- and 20th-century psychiatrists (7).

In recent times, empirical studies in the areas of clinical psychiatry and psychology and translational neuroscience have emerged in large numbers, but using classical psychopathological paradigms lacking any updating or adequacy. It was the scarcity of the results of these studies that spurred a resurgence of a broader way of investigating in psychopathology that should always include a thorough analysis of concepts and the most appropriate epistemology to be used.

In this resarch topic, we have achieved the objectives we have set by bringing together several review and perspective articles that critically analyze the various models used in psychopathology and propose new ways to research in this field. Several original studies were also included, some of which also challenge the classical psychopatological concepts.

## Author Contributions

DT contributed to the conception and design of the article. DT and EC contributed to the manuscript revision, reading, and approval of submitted version.

## Conflict of Interest

The authors declare that the research was conducted in the absence of any commercial or financial relationships that could be construed as a potential conflict of interest.
